# Land degradation: Addressing the vulnerability of local people through the lens of transformative change

**DOI:** 10.1016/j.heliyon.2024.e37891

**Published:** 2024-09-13

**Authors:** Ruxandra Malina Petrescu-Mag, Tibor Hartel, Kinga Olga Reti, Cornel Mocanu, Ioan Valentin Petrescu-Mag, Vlad Macicasan, Dacinia Crina Petrescu

**Affiliations:** a Faculty of Environmental Science and Engineering, Babes-Bolyai University, 30 Fantanele Street, 400294 Cluj-Napoca, Romania; b Gembloux Agro-Bio Tech, University of Liège, 2 Passage des Déportés, 5030 Gembloux, Belgium; c Doctoral School “International Relations and Security Studies”, Babes-Bolyai University, 1 M. Kogalniceanu Street, 400084 Cluj-Napoca, Romania; d Doctoral School of Engineering, University of Oradea, 1 Universităţii Street, 410087 Oradea, Romania; e Department of Environmental Engineering and Protection, Faculty of Agriculture, University of Agricultural Sciences and Veterinary Medicine of Cluj-Napoca, 3-5 Calea Mănăștur Street, 400372 Cluj-Napoca, Romania; f Faculty of Business, Babes-Bolyai University, 7 Horea Street, 400174 Cluj-Napoca, Romania

**Keywords:** Participatory, Community, Agriculture, Windbreaks, Resilient irrigation system

## Abstract

Land degradation (LD) is driven by many factors resulting from the intricate interplay between natural and socio-economic systems, which adds dynamism and complexity to this phenomenon. The study highlights LD as a source of social vulnerability in the Baragan Plain (Romania), often called the “granary of Europe” due to its century-long history of industrial crop production. We explore the community's perceptions of vulnerability due to LD and the community-based solutions to sustainable transformations through governance using a community-based causal-effect analysis (CBCEA). CBCEA is a participatory approach that uses systems thinking, engages key informants, and generates qualitative causal-effect diagrams to illustrate the system structure. Two workshops with local key informants revealed their views on the direct and indirect causes and effects of LD, strategies they proposed to reduce the community's vulnerability, and the conditions for making the agricultural land decision-making integrative, inclusive, adaptive, and participatory (IIAP). IIAP decision-making was considered a key to transformative governance. Key informants identified “Windbreaks construction” and “(Resilient) Irrigation system” as two effective, context-specific measures to address the causes and effects of LD. We advise caution when implementing the “Irrigation system” measure, as it may risk constraining the system to an undesirable state, commonly referred to as a “trap”.

## Highlights

• Land degradation (LD) is as a source of local people vulnerability in Baragan Plain, Romania.
• The CBCEA illustrates community's understandings of LD.
• The direct and indirect causes and effects of LD are identified.
• Community-based solutions toward transformative governance are presented.
• “Windbreaks construction” and “(Resilient) Irrigation system” can counteract LD.

## 1 Introduction

The land is a vital arena for ecosystems and nature conservation and a highly sought-after resource that supports various human needs [1,2]. The United Nations Convention to Combat Desertification (2022) signals that we have already degraded around 40 % of the land, directly affecting half of humanity and threatening almost half of the global GDP (US$44 trillion). How will this land sustainably feed and shelter 9.8 billion people in 2050 and beyond? The increasingly extreme climatic variations substantially amplify the social and environmental drama [3,4] of this question.

Severe land degradation (LD) affects developing and developed countries alike on all continents. In Europe, the Mediterranean region is recognized as a hotspot for desertification [5]. In central and eastern Europe, LD is documented in various forms, from extreme fragmentation of farmland ownership [6], changes in tillage practices, and other agricultural technologies [7] to deforestation [8]. LD was investigated primarily in Romania from land cover change, water variability, and climate change [[9], [10], [11]] and less from a social perspective [12,13].

LD is part of a complex system, and therefore, social and environmental (including ecological) processes should be captured under the lens of “systems thinking” to understand it [14,15]. System dynamics is a methodological approach that uses systems thinking to identify the relationships between causes and effects, also called causation, and understand how the system works [14,15].

The present study aims to advance the field of transformative change by addressing critical gaps in understanding the evolving dynamics of land degradation (LD). Through participation in local communities, this research captures the nuanced and context-specific causes and effects of LD, which are often overlooked in more extensive studies. This paper addresses the complex interconnections between various social and natural systems components, providing a holistic perspective on LD, and emphasizing the importance of system thinking. The study uses the community-based causal-effect analysis (CBCEA) to identify actionable and contextually relevant measures capable of driving transformative change in LD management. One novelty of this research is its use of CBCEA, which prioritizes systems thinking potential among key informants and enables the creation of qualitative causal-effect diagrams to describe the system structure comprehensively.

Furthermore, the study offers straightforward policy suggestions to inform sustainable land management strategies locally and nationally. Through these contributions, the study aims to fill existing knowledge gaps and provide a comprehensive understanding of the factors that influence 10.13039/100031136LD, ultimately supporting more effective and sustainable land management practices. To achieve these goals, the study is guided by the following key objectives.

i) To identify the direct and indirect causes and effects of LD in the study area through community participation and the application of CBCEA analysis.
ii) To develop qualitative causal-effect diagrams that provide a comprehensive understanding of the system structure related to LD, incorporating the perspectives of key informants.
iii) To identify actionable and contextually relevant measures for sustainable land management to facilitate transformative change in agricultural land decision-making.

To further explore and address these objectives, the study posed several exploratory questions (EQ) to gain a more comprehensive understanding of the community's perspectives on causes, effects, solutions, and factors that can facilitate transformative change in management of LD.

(1) “What is the community's shared understanding of the direct and indirect causes of LD in the study area?”
(2) “What is the community's shared understanding of the direct and indirect effects of LD in the study area?”
(3) “What are the measures (solutions) proposed by the community to reduce the vulnerability to LD in the study area?“, and
(4) “What must happen for agricultural land decision-making to become transformative?”

## 2 Navigating vulnerability through transformative pathways with CBCEA - A comprehensive literature review

Multiple factors contribute to the degradation of agricultural land, with the socio-economic and natural ecosystems interacting as interrelated systems of land degradation [16] that add complexity and dynamism. The land is prone to degradation due to farming techniques, over-cultivation, the use of pesticides [17,18], urbanization, deforestation, and extreme weather events [19]. Agricultural LD becomes a source of vulnerability, understood as the system's susceptibility to experience harm due to socio-economic and environmental factors and their interactions [20]. LD is caused by direct and indirect economic, social, and environmental factors [21]. LD reduces environmental services and agricultural productivity [22], affecting everyone to some extent, through food insecurity, climate change, higher food prices, and environmental hazards [23,24].

Many definitions are used in the scientific literature, often with different disciplinary-orientated meanings [25], to reveal what LD is. Gibbs and Salmon [26] consider that understanding “degradation” is challenging because it is represented under a variety of conditions (e.g., desertification, erosion, salinization, invasive species) or only as a subset of these conditions. However, there is a consensus that degradation is due to human activity. We adopted the definition of LD included in the IPCC report [27] mainly due to its social importance. Thus, LD is “a negative trend in land condition, caused by direct or indirect human-induced processes including anthropogenic climate change, expressed as a long-term reduction or loss of at least one of the following: biological productivity, ecological integrity or value to humans” [27].

This study examines Facaieni village in Ialomita county, Romania, where agricultural intensification has been a prominent feature for nearly a century. LD impacts land productivity and has social consequences in the study area. Ialomita is part of the Baragan Plain, often called the “granary of Europe”. The origins of this appellation go back to 1938. That year, there was a world wheat crisis due to adverse weather conditions. Romania achieved its largest wheat export in history at that time, exporting over three million tons of wheat [28]. Vorovencii [29], who monitored the risk of land degradation in the Baragan Plain, highlights that there is currently a significant fragmentation of land ownership, with many individual farms engaging in subsistence agriculture. Consequently, agricultural land is being converted into various categories, including abandoned land, with a growing susceptibility to degradation.

It should also be highlighted that the impacts of LD occur in downstream areas of the biosphere due to the interconnectivity between ecosystems on different scales [30]. Therefore, sustainable land management must be relevant across local and global scales [31]. Henry et al. [30] believe that a successful upscaling from a local area requires a common body of knowledge on sustainable land practices. This knowledge also includes tools for comparative assessment, selection, and fine-tuning sustainable land management practices for various ecological, economic, social, and cultural conditions [30]. Participation of local stakeholders (farmers, policymakers, investors, consumers, and researchers) facilitates the adoption of sustainable land practices [32].

Recognizing the importance of engaging local land stakeholders, we focus on system science, particularly CBCEA, a participatory method derived from community-based system dynamics (CBSD), that aims at understanding a problem by promoting systems thinking to solve it [33,34]. Unlike CBSD, CBCEA stands out for its simplicity. It offers a straightforward and intuitive approach to visualize the interconnections among variables within a complex system [35]. The CBCEA approach reveals the perceptions of the community about how people can influence, in our case, sustainable land management. The community is formed by those who fundamentally engage in building a shared understanding [33]. Therefore, CBCEA relies on the perceptions, knowledge, and experiences of community members who co-create new knowledge [36]. These collaborative approaches become “transformative” when they encourage participants to present transformative strategies, action plans, and policy pathways [37]. CBCEA is an appropriate tool to achieve transformative change because, in transformative processes, the “classical” difference between expert and stakeholder knowledge is “unhelpful and outdated”; also, it is considered that the presence of different stakeholders with various experiences and knowledge fields is legitimate [38].

Transformative change is seen as game-changing shifts [39] or “fundamental, system-wide reorganizations across technological, economic, and social factors, including paradigms, goals, and values” [40] *ap.* [39]. This implies that society's institutions, norms, and practices must be reconfigured [41,42]. Reconfiguration is necessary because it reshapes the goals, attitudes, values, and behaviors critical to investigating the direct and indirect causes of biodiversity loss [39], and in the present case, of LD. The transformative change is mandatory to address LD, as current structures represent indirect causes of LD [43]. Different authors mention that these indirect causes can be demographic (e.g., migration), sociocultural (e.g., consumption patterns), economic (e.g., lack of market), or related to different events (such as pandemics and conflicts), institutions and governance, and are supported by values, behaviors, and societal goals [44,43]. The indirect factors serve as the underlying drivers of the direct causes of LD, such as pollution, deforestation, or urban sprawl [[45], [46], [47]]. While direct and indirect causes are local and context-dependent, making it difficult to extrapolate from one case study to another, transformative governance, crucial for sustainability in social-ecological systems [48], provides a framework to respond, manage, and generate regime changes [43,49], thereby addressing these unique and varying circumstances.

The existing literature provides a comprehensive understanding of LD, emphasizing its multifaceted nature driven by various socio-economic and environmental factors. The interrelated systems of natural and socio-economic ecosystems contribute to the dynamism and complexity of LD, with significant implications for agricultural productivity and social well-being. While previous studies have extensively explored the direct and indirect causes of LD, including human activities such as deforestation, urbanization, and the use of pesticides, as well as natural events such as extreme weather, there remains a critical gap in integrating local people's perceptions into sustainable land management practices. Furthermore, although transformative change is recognized as essential for addressing LD, there is limited empirical evidence on how community-based approaches, particularly CBCEA, can facilitate this process by capturing context-specific insights and fostering systems thinking. This study aims to fill these gaps by employing a participatory methodology to engage local stakeholders, thus providing a deeper understanding of the nuanced dynamics of LD and identifying actionable measures for transformative change in agricultural land decision-making.

## 3 Methodology

### 3.1 Land degradation in the study area

The study area is in the southeastern part of Romania, in the eastern division of the Romanian Plain. More precisely, it is the Facaieni village of Ialomița county (the county extends over 4453 km^2^, representing 1.9 % of the total area of the country) [50] (Fig. 1). Facaieni is the second largest rural town in Ialomita county, with 5304 inhabitants.

**Fig. 1:**
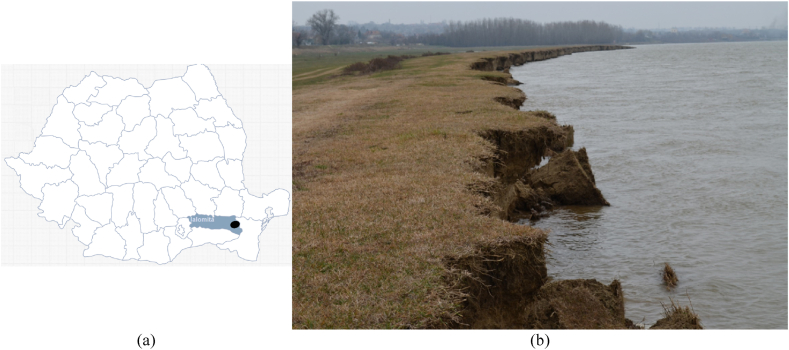
a) Facainei study (black dot) in Ialomita county (blue background); b) Riverbank erosion in Facaieni study area, 2023. (For interpretation of the references to colour in this figure legend, the reader is referred to the Web version of this article.)

Source: a) created by the authors using Simplemaps [51]; b) Authors' personal archive (Cornel Mocanu)

In Romania, the agricultural area represents 56 % (13,3 million ha) of the country's territory (23,8 million ha), of which the largest part consists of arable land (62,5 %), followed by pastures (22 %) (Ministry of Agriculture and Rural Development, 2015, https://www.madr.ro/docs/agricultura/agricultura-romaniei-2015.pdf). In Ialomita county, the agricultural area represents 84 % (374 495 ha) of the total county's territory (445 289 ha), of which arable land occupies 94 % (352,146 ha) [52]. The Ialomita Floodplain covers 76 % of the county's surface, and the eastern part of the county belongs to the Danube Floodplain (Balta Ialomiței, 15 %), which makes the area very attractive for agricultural activities [53]. Regarding soil types, we find Mollisols (Kastanozems), with a predominance of Chernozems, Clay-illuvial Chernozems, and Cambic Chernozems, which occupy the largest areas [53].

The county is one of the most deficient in water at the country level. In 1990, immediately after the fall of communism, more than 86.2 % of the surface of arable agricultural land (947,600 ha) was managed using irrigation systems; by 2010, the percentage had decreased to 6.9 % [29]. The absence of irrigation systems results in climate-dependent agriculture, heavily affected by prolonged and excessive droughts. Soil crusting, land aridity, decreased soil fertility, and soil salinization are consequences of the lack of irrigation systems in the Baragan Plain [29].

**Fig. 2:**
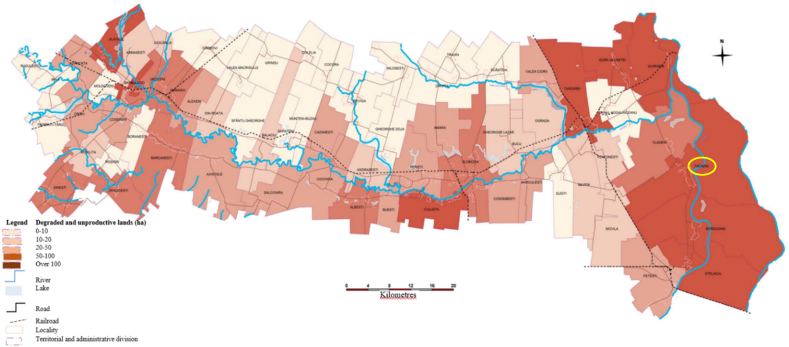
Degraded and unproductive land in Ialomita county (Facaieni is marked in yellow). (For interpretation of the references to colour in this figure legend, the reader is referred to the Web version of this article.)

Approximately 115,011 ha of land in Ialomita County are affected (Fig. 1, Fig. 2) by various factors that cause LD, such as poor and very poor mobile phosphorus provision (27.8 %), soil salinity (9.4 %), extremely low to low humus reserves, irrational use of chemical fertilizers and pesticides, inappropriate waste storage, the vulnerability of most administrative-territorial units in the county (83 %) to nitrate pollution, and the existence of 10 potentially contaminated sites from the oil industry [53].

Source [54].

### 3.2 The qualitative modeling processes

#### 3.2.1 The community-based causal-effect analysis

CBCEA is a participatory method for system dynamics modeling that involves the community in understanding and changing systems. CBCEA generates causal-effect diagrams [33] using computer simulations (Vensim PLE 9.3.3 software) that express the relations between variables [55]. The simulation models help visualize the connections between variables and, thus, identify measures to change that structure that can, ultimately, influence the whole system [[56], [57]].

Diagrams help reveal system dynamics through visual representation of the causes and effects of LD and their interrelationships [58]. Arrows were used to indicate the relationship between variables. Polarity signs (”+“and “- “) of variable relationships facilitate understanding of the structure of the system and how a specific outcome can be further influenced [58]. The “+” sign on the arrowheads shows that a variable positively relates to another one (e.g., increasing one leads to increasing the other). The causal relationship of these variables helps to determine more “upstream” influences [36]. Practically, the network of positive and negative feedback and all dynamics arise from the interaction of these diagrams.

#### 3.2.2 Selection of key informants and research strategy for the CBCEA

The selection and participation of key informants and the entire research adhered to strict ethical standards, as agreed upon by the Scientific Council of Babes-Bolyai University (which is the Ethics Approval Committee). The manuscript, referencing Research Ethical Approval 92/January 06, 2023, underscores a commitment to ethical practices. The participants were informed about the aim of the research and the option to withdraw at any point during the interview. The participants did not receive any form of payment or other rewards. All data presented in the manuscript have been anonymized to protect the identity of the participants. Volunteer participation was secured by obtaining written informed consent from each participant, highlighting the importance of explicit consent in the publication of their data. This comprehensive approach ensures that the manuscript meets ethical standards, as explicitly outlined in the Ethics and Consent section.

Key informants in this research refer to people living in the study area with deep connections to the local social and environmental dynamics through direct experience or narratives passed down by local elders. They are interested in finding solutions for sustainable agriculture. If the selection process of the key informants is superficially designed, key informants may only have a consultative role rather than an inclusive role capable of providing genuine deliberations [59,60]. To avoid this and ensure the active participation in the workshops and a diversity of perspectives, we selected 15 key informants from various occupational areas (e.g., agricultural mechanic, agronomist engineer, pensioner, teacher, administrator, welder, and several day laborers in agriculture). The average age was 48.26 years. One of the main considerations in their selection was to create a panel with a combination of people who could offer information on the challenges caused by LD within their community. Burns [61] highlights the trade-offs in terms of the homogeneity of participants between Participatory Action Research (PAR) and Systemic Action Research (SAR). There are advantages and disadvantages to PAR and SAR. Still, because of the specificity of the present case study (e.g., we scrutinized the existing interests, and there was no prominent source of power asymmetry), we opted for SAR.

We always tried to reveal the diversity of the participants’ views and to give space to those voices at risk of being diminished (for example, economically disadvantaged people, such as day laborers). From the beginning, we tried to create a safe communication space for each workshop through several ice-breaking exercises (e.g., “Find 5 things in common”; “What if exercise”, and “Word association”/“I say one word, you say two”). Additionally, we introduced “workways” [62], which outlined principles such as confidentiality, active listening, empathy, tolerance for differing opinions, the understanding that agreement is not necessary on every point, and the freedom to leave the workshops. The biases of the research team were mitigated using the “handing over the stick” (pen) approach [63].

Within the case study on LD in Facaieni, Baragan Plain, the objectives of the qualitative modeling process were the following.

(a) To reveal the causes and effects of LD based on the key informants' understanding.
(b) To reveal what measures (solutions) key informants consider influencing the elements (causes and effects) of the diagrams.

After preliminary steps, participants were asked to mention the direct and indirect causes of LD (in the first workshop, Table 1) and then to think about the type of relationships between causes, effects, and LD (in the second workshop, Table 1). Researchers facilitated discussions to achieve consensus. Using this information, researchers drew the diagrams in Vensim and presented them to the participants for validation (in the second workshop, Table 1). Next, a set of hypothetical questions (“what if”) was used to explore possible measures to influence the causes and effects of the LD, as illustrated in the diagrams (workshop 2, step 5, Table 1): “(1) What would happen if LD in your region no longer existed?“; “(2) What should happen for things to go well?“; “(3) If you knew there was a solution to LD, what would this be?“. Key informants were asked to refer to the diagrams while answering these questions. However, because there were many diagrams, it would have taken considerable time to identify the measures for each one. Therefore, we asked participants to identify, among the elements of each diagram, those over which they believe they can control (they have the political and economic power to modify them). The next step was to identify at least one common measure that could influence the elements of the diagrams. Based on the answers, the facilitators synthesized the measures.

**Table 1:** The development of the workshops.

Workshop 1	Workshop 2
**Part I. Preparatory steps** **1.** Welcome and general presentation of the workshop, participants, etc.; **2**. Creation of a safe communication space (ice-breaking exercises and the ways of working); **3.** Definition of their “community” by participants; **4.** Introduction to systems thinking, familiarization of key informants with system concepts and language, and facilitation of systems thinking around factors that could influence agricultural activity in their area; **5.** Definition of the problem with the participants by brainstorming the main challenges related to their agricultural activity (e.g., productivity, weather conditions, support from public authorities). Photo elicitation was also used [64]. **Part II. Direct and indirect causes and effects of LD** **6.** The participants brainstormed key issues that they considered to influence LD (the causes of LD). First, they were asked to think of direct causes and then indirect ones. Second, they were asked about the direct effect of LD, followed by the indirect effects; **7.** Consensus building check: brief discussion of the identified variables; **8.** Reflection in workshop 1.	**Part III. The LD systemic model** **1.** Review of main aspects discussed during the first workshop; **2.** Key informants were asked to think about the nature of the relationship between the determinants identified in workshop 1, and then include polarity markers (±) to determine whether the connections are positive or negative; **3.** Researchers draw causal-effect diagrams; **4.** Revision of the model by the participants (validation of the model). **Part IV. Strategies** **5.** Brainstorm ways to counteract the causes and effects of LD; **6.** The researchers synthesized the measures proposed by the participants in two strategies; **7.** Consensus building check with participants on strategies. **Part V. The IIAP decision-making for agricultural land** **8.** Group interview (four questions were asked) to find out how governance could become IIAP 9**.** Reflection exercise in workshop 2.

Next, following the assumption of Visseren-Hamakers et al. [43] that governance will become transformative if it is simultaneously integrative, inclusive, adaptive, and participatory (IIAP), we asked one question for each characteristic of transformative governance (see workshop 2, Table 1). Key informants were asked to consider mainly the indirect causes that formed the diagrams.

For the *integrative* character, the question was: (a) “What measures could local/central public authorities take so that various aspects related to agricultural land health are integrated in a way that ensures the creation of value for your community and the results are beneficial for all interested parties (farmers, non-farmers) in the long term? (An explanation was added: “What measures must the authorities take to improve the condition of the land so that the entire community benefits?").

For the *inclusive* character, the question was: b) “What measures do you think central/local public authorities should take so that you can play/participate in the well-being of your community with benefits for agricultural land?”

To capture the *adaptive* character, we asked: (c) “How could local/central public authorities be mobilized/determined/stimulated to respond (through various normative acts, programs, actions) to the changes caused by LD”? (An explanation was added: “Community members must adapt, and the authorities must help them. How do we determine the authorities to help them adapt?“)

For *participatory* governance, we addressed the question: (d) “What could be done to make the central/local public authorities consider the opinions and the position of the community members regarding LD? How could authorities include the most disadvantaged?”

The two workshops (of about 2 h each) were facilitated by three researchers (they are the authors, and one of them is also a community member). The workshops were recorded in audio/video, and the text was transcribed verbatim using VatisTech software. Written informed consent was obtained from the participants. A brief presentation of the steps of the workshops is provided in Table 1.

## 4 Results

### 4.1 Preparatory steps of the CBCEA workshops

One of the ice-breaking exercises included the “if questions” and aimed to reveal what people would like for the welfare of their community. We asked: “If I were the goldfish, what would be two wishes that you would like me to fulfill related to your community or the activity you carry out here?” The answers were mainly related to employment opportunities and investments in the region. Others talked about the need for utilities, more precisely, the introduction of gas.

**Fig. 3:**
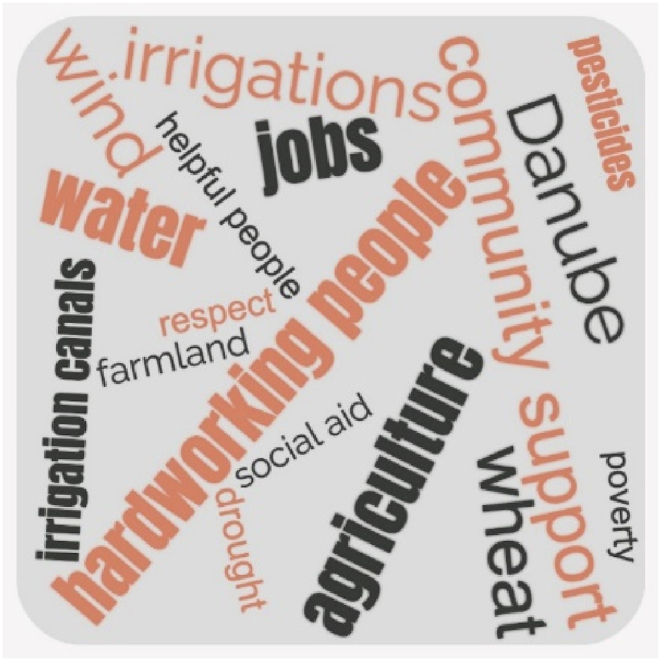
Word cloud defining the community.

The game “I say one word, you say two words” together with the strategy “handling over the stick” (a pen in our case) allowed everyone to express their opinion and was used to highlight the keywords that define the community. Fig. 3 illustrates the words spoken by the participants that reflect the characteristics of the community to which they belong.

Five photoswere used to illustrate various types of agricultural LD in the country (Fig. 1A, Annex). The definition of the problem resulted from contrasting the current situation of agricultural land with the one that existed 30 years ago.

### 4.2 Direct and indirect causes and effects of LD, Systemic model of LD, and the community-suggested measures (solutions)

To respond to the first and second EQ, the participatory process revealed the direct and indirect causes and effects of LD. Table 2 highlights the variables included in each causal-effect diagram as identified by key informants (the diagrams were initially created in Vensim, and them edited for clarity in Word, Microsoft Office). Key informants identified 12 causal-effect diagrams. The diagrams also included the two measures suggested by the participants to address the causes and effects of land vulnerability.

**Table 2:**
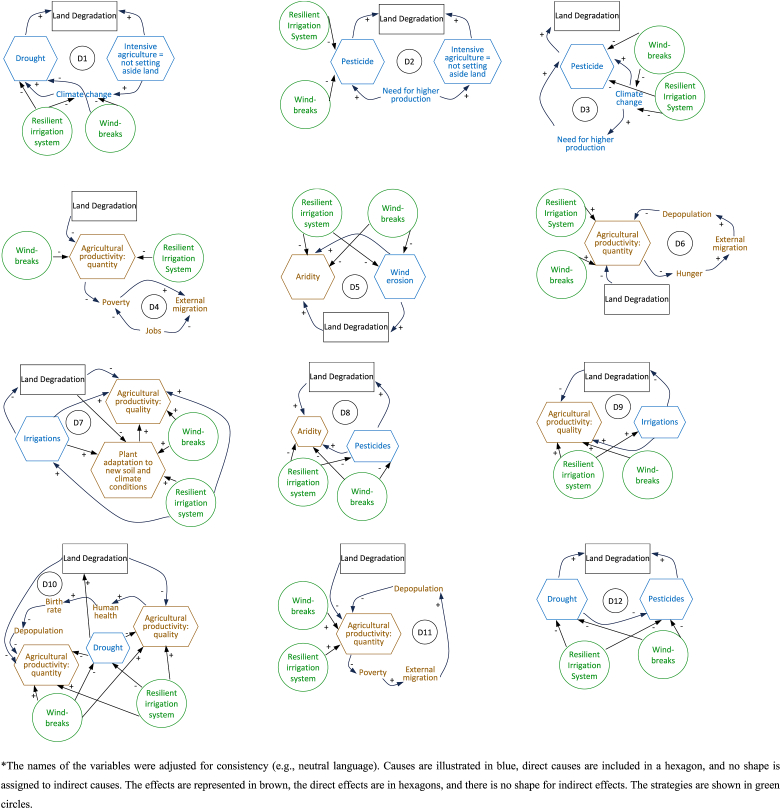
The causal-effect diagrams and their relations to “Windbreaks” and “(Resilient) Irrigation System” strategies.

∗The names of the variables were adjusted for consistency (e.g., neutral language). Causes are illustrated in blue, direct causes are included in a hexagon, and no shape is assigned to indirect causes. The effects are represented in brown, the direct effects are in hexagons, and there is no shape for indirect effects. The strategies are shown in green circles.

Understanding the causes and effects of LD identified by participants requires further explanations. Key informants saw “Pesticides” as a direct determinant of LD. They mentioned that large landowners and farmer associations (“Associations, big landowners” an indirect cause of LD) are the main responsible for the overuse of pesticides. In contrast, small farmers use fewer pesticides and more natural fertilizers. For migration, they insisted on mentioning only “External migration”, mainly to Italy. “Intensive agriculture-not setting aside land” referred to maximizing agricultural production by not applying good farming practices. Specifically, it did not set aside the land due to the need for larger amounts and the existing weather conditions.

When asked what causes and effects of LD they considered a source of community vulnerability, key informants could not discriminate between them and considered all variables a source of vulnerability. The direct and indirect causes and effects of LD (forming the diagrams) were then included in a text box to facilitate the next step. Respondents were asked to think about possible solutions or measures that could primarily counteract those causes and the effects when answering questions 1–3 [”(1) What would happen if LD in your region no longer existed?“; “(2) What should happen for things to go well?“; “(3) If you knew there was a solution to LD, what would it be?“]. The facilitators synthesized their answers into two main measures to counteract the causes and the effects of LD (EQ (3)): “Windbreaks construction” and “Irrigation system”. Considering the need for “Resilient irrigation system”, we use this name in Table 2.

### 4.3 The IIAP decision-making process for agricultural land

**Fig. 4:**
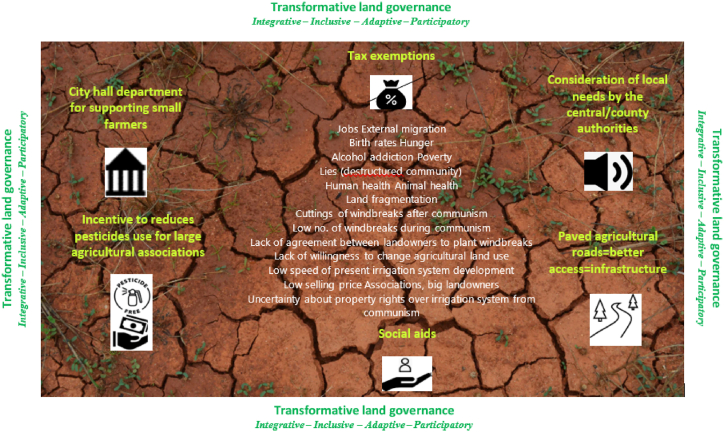
Solutions of key informants' for IIAP decision-making (the LD's indirect causes in the centre).

For the last step of workshop 2 (Table 1) (the group interview), key informants were asked to answer four questions, mainly thinking about the indirect causes and effects of land degradation (EQ (4)). The responses aimed to reveal how agricultural land decision-making could become IIAP. As mentioned in the Introduction section, the focus on indirect causes and effects was motivated by the fact that transformative change should mainly address the indirect causes of a problem, according to Visseren-Hamakers et al. [43]. Fig. 4 illustrates the solutions offered by key informants to achieve IIAP decision-making for agricultural land. They refer to “Tax exemptions”, “Social aids”, “Consideration of local needs by the central/county authorities”, “City Hall department for supporting small farmers”, “Incentive to reduce pesticides use for large agricultural associations”, and “Paved agricultural roads = better access = infrastructure”.

## 5 Discussion

### 5.1 Preparatory steps of the CBCEA workshops

The study examines the impact of LD on vulnerability in the Baragan Plain through qualitative research. The exercises “What if” and “I say one word, you say two” (workshop 1, steps 2, and 3, respectively, Table 1) depicted a community dedicated to agriculture, mainly to cereal cultivation., They showed a strong sense of place, mentioning terms such as “Danube”, “village”, or “neighborhood”. Despite shared interests and joint agricultural activities, concerns about the well-being of the community related to crop productivity arose. The lack of irrigation and use of pesticide use were prominent concerns, with a notable detachment from guilt, which was attributed cautiously to agricultural associations, even among participants affiliated with them.

Facaieni shows tight-knit interpersonal relationships, fostering a community where mutual assistance prevails despite having many inhabitants. The town hall serves as a cohesive force. Residents are viewed as “respectful” and “hard-working”, participating in reciprocal help, from borrowing goods to helping with fieldwork and communal tasks. This contrasts sharply with Transylvania, where Hartel et al. [65] noted rising individualism, mistrust, and self-perceived poverty. Key informants also mentioned that the community faces many shortcomings (“poverty”, “social aid”, and “lack of jobs” were some of the keywords used to define that state). Participants attributed them to the decline in agriculture, which they felt more strongly after the fall of communism. The fall of communism is seen as a pivotal moment, resonating with the broader challenges faced by former communist nations during their transition to capitalism [66].

Following MacQueen et al. [67] and Mormont's [68] understanding of community, we highlight that the investigated community can be viewed in two ways: as a community that belongs to a particular geographical space and as a social representation constructed on shared interests and perspectives and joint actions that members can enjoy by participating in networks of relationships.

Furthermore, to identify the problem (workshop one, step 3, Table 1), key informants remembered how the situation was 30 years ago. One participant reflected that “Let's compare the 1990s and the present. The situation has worsened a lot. We have a type of sandy soil here, and even if it rains, everything dries up in 2 h when the wind blows.” To focus the discussion on agricultural land, several photos that illustrate failed crops and land with low resilience due to desertification (sandy land), aridity, and intensive grazing were presented to participants (Fig. 1A, Annex). Photo-elicitation (step 6, workshop 1, Table 1) also had the role of easing the communication between facilitators and key informants. Although not all photos provided new information, photo-elicitation could still evoke new meanings and insights into the researched problem for the interviewee [[69], [70], [71]]. For example, for key informants, LD was perceived as a decrease in agricultural productivity, probably because the Baragan Plain is known as a farming region, and many people live off agriculture. This connection is not surprising because, in another study, Romanian farmers perceived soil productivity as the most important soil function, closely related to the monetary value of agricultural activity [72].

### 5.2 Direct and indirect causes and effects of LD, the Systemic model of LD, and the community-suggested measures (solutions)

In system dynamics modeling, the most important variables of LD, as agreed upon by the majority of key informants, were included in the diagrams (workshop 1, steps 7 and 8; workshop 2, steps 2–4; Table 1) to illustrate their interactions with each other [73]. The number of causal-effect diagrams indicates the complexity of the system. In addition to the variables illustrated in the diagrams, other variables influenced the system (e.g., “Alcohol addiction”, “Land fragmentation”, “Associations, large landowners”), pointing to the complex web of causes and effects of LD.

Although we know from the diagrams (D1-D12) that the constituent factors have a direct impact on LD, other influential factors can only be identified by exploring other variables (e.g., “Cuttings after communism”, “Low selling price”). D1-D12 helped us understand the non-linearity of the cause-effect relationships of LD and how small changes could have significant effects and make the system unstable [36,58].

Diagrams represent the mental model of LD of key informants in the study area, constructed from the causes and effects of LD and assumptions about their relationships as identified by the participants. D1 shows that when drought (considered by key informants as the dryness of the land, not the lack of precipitation) and intensive agriculture are more prominent, so is LD, and more intensive agriculture is used, the more visible the effects of climate change are, which, in turn, increases the manifestations of drought. Interviews with laypeople in Romania revealed a similar perception, where farmers – regardless of whether they were involved in intensive agriculture or large-scale farming – were often viewed as the “villains” of climate change [74]. D2 demonstrates that the community's need for higher agricultural production influences the use of more pesticides and the intensive farming practices that directly influence LD. D3 and D8 illustrate that interviewees have a widespread perception that the more present the effects of climate change are, the higher their need for agricultural production and the higher the quantity of pesticides used. Additionally, a higher quantity of pesticides exacerbates the aridity in the area. In general, the intensive use of agrochemicals has often been cited in the climate change literature as an adaptation strategy [75,76].

D4 demonstrates how a decrease in job availability translates into more external migration and poverty, which also influences migration. In D5, we show that more wind erosion leads to more LD, increasing aridity. The quantity of production plays a central role in D6; a decrease in quantity is translated into more hunger, which causes more community members to migrate to other countries, thus affecting the size of population. Therefore, as more people leave the community, more depopulation occurs. D7 and D9 highlight the role that key informants believe irrigations play in the quality and quantity of their agricultural products.

Furthermore, fewer irrigations will cause plants to be unable to adapt to new soil and climate conditions and significantly degrade the land, and more LD will cause a decrease in the quality and quantity of the products. More drought, less quality and quantity. Key informants agreed that depopulation leads to a reduction in production, as fewer people are available to work the land. When exploring D10 and D11, it is evident how human capital is influenced by the quality of the agricultural products that people consume. Drought influences both the quality and quantity of agricultural products. Consistent with these assumptions, the literature on drought reports various health effects for people, with additional social and economic consequences that increase their vulnerability [77]. For example, Bryan et al. [78] associate drought with worsening human health through reduced water quantity, compromised hygiene, food security, and nutrition. D12 shows that during drought, people use fewer pesticides. Next, more pesticides and more drought result in more LD.

Workshop 2, step 5 (Table 1) revealed participants’ suggestions on actions to take to counteract the identified causes and effects of LD. The researchers converted their responses into two measures “Windbreaks construction” and “(Resilient) Irrigation system”. Key informants considered field protection systems, especially windbreaks, to be a stringent need. According to Smith et al. [79], windbreaks are an agroforestry practice consisting of linear plantings of trees and shrubs integrated into the agricultural landscape that brings environmental, economic, and social benefits. In addition, financial efforts should be directed towards irrigation canals. The participants mentioned an ongoing European project focused on irrigation infrastructure and expressed their hope for its successful implementation. Facaieni is close to the Danube and other waterways, and this resource must be used with attention to waterlogging and degradation of ground and surface waters [80,81]. Therefore, irrigation must consider short-term versus long-term perspectives, externalities (e.g., taxes, but they are challenging to implement), risks, and uncertainty [82]. Side effects of irrigation systems are often reported, such as the salinity level in the drainage water [83], conflicts between users [84], and the presence in drainage water of minor elements (selenium) that are harmful to birds and fish [85].

Water management for agriculture is related to natural resources, food production, and rural development [86]. Therefore, the interests of different stakeholders affected by water allocation should be carefully considered [87]. Thus, water demand must be met by diversifying the supply sources in combined systems [86] and using available water supplies more efficiently. Farmers should also reevaluate what crops to plant and in what area [88]. Ialomita county is highly dedicated to cereal cultivation, and attention should be paid to the observations made by Manjunatha et al. [89]. They consider crop specialization a good short-term option due to financial profitability. Still, in the long run, it could have negative environmental consequences, such as loss of soil fertility, water logging, and salinity. The vulnerability of irrigation systems lies in their high centrality, leading to power asymmetry and detailed coordination of water use, according to Janssen et al. [90].

Consequently, the irrigation system to which the key informants referred must encompass the characteristics of a “Resilient irrigation system”. However, the “Resilient irrigation system” is a work-in-progress concept in irrigation science and is highly contextual. It has three main features: capacity to absorb (a strong absorptive capacity leading to persistence), capacity to anticipate (implies transformational responses in front of unexpected changes), and adaptive capacity (means incremental adjustments and adaptation) [91]. All these capacities are connected to systems (infrastructure, river basins), institutions (management, regulations), and agents (individuals, organizations) [92]. If the system, institutions, and agents are not designed and functioning properly, the “Resilient irrigation system” becomes unattainable. Considering the local social, economic, and political determinants, the “Irrigation system” measure should be approached with caution, as it can become a “social trap”. The “social trap” occurs when individuals seek immediate positive outcomes, ultimately leading to negative long-term consequences for both individuals and society [93].

The efficiency of water use can also be improved by implementing windbreaks, which reduce the impact of wind speed [94]. The reviewed articles [94,95] do not report the concerns of the respondents about the impact of windbreaks on the availability of water resources.

Key informants identified wind erosion as a direct cause of LD. In practice, nutrients such as organic carbon and phosphorus from the topsoil are blown away [96]. Wind erosion affects not only agricultural productivity but also air quality and human health. Smith et al. [79] conducted a review study on windbreak adoption in the U.S., covering the 1949–2020 period, and found that windbreaks on agricultural lands are mainly used for indirect economic benefits, such as snow control, wind protection soil, and erosion control. This is followed by direct agricultural benefits (e.g., increased crop through the creation of a microclimate that can lead to higher productivity [97]) and intrinsic values (wildlife habitat, aesthetics). Irwin et al. [98], who investigated the attitudes and perceptions of Irish farmers about planting trees on their land, found that they most prefer planting trees on their farms. However, the change in land use to forest is a significant obstacle to tree planting, which is consistent with what we found in our qualitative research. In contrast to the results of the present study, windbreaks were negatively perceived by more than half of Kyrgyzstan's farmers, mainly due to their concern about a decrease in productivity caused by the shade of crops by trees [94].

Considering also factors that influence “Land fragmentation”, “Lack of agreement between landowners to plant windbreaks”, and “Lack of willingness to change agricultural land use”, participants stated that a solution to combat “Wind erosion” would be the enforcement of law for mandatory windbreaks. They suggested that by this law, community members should be compensated with a sum of money to change the destination of agricultural land to windbreaks. They disagreed on expropriation, saying the land must remain in their ownership. Similarly, unresolved ownership rights are one of the main obstacles to the planting of agricultural windbreaks in the Czech Republic [99]. In the present study, key informants would agree to participate in the planting activity if the procedures were simple and bureaucracy disappeared, which was invoked several times as a significant impediment.

In a 2012 study [100], the total agricultural area that must be analyzed for the location of forest shelterbelts in the Romanian Plain was 2,806,989 ha. In Ialomita county, windbreaks should have been planted on approximately 50 ha, but only 24 ha have been planted [101]. It must be underlined that a law (Law No. 289/2002) regarding windbreaks was recently modified in Romania (Ordinance No. 36 of August 31, 2022). One of the most expected changes is that farmers are no longer obliged to cede to the state the area of land they forest, as was the case until now.

In 2022, Romanian legislation changed, allowing farmers to retain ownership of land used for windbreaks protecting crops. Previously, such land automatically became state property. The EU now grants 456 euros per hectare for 20 years to support forested areas. Ordinance no. 36/2022 [102] designates specific regions, including the Romanian Plain and the Danube Meadow, eligible for windbreaks due to frequent drought. Key informants were unaware of these changes, which signals the need for local information campaigns and technical support to navigate bureaucratic procedures for windbreak implementation.

Practically, the key informants advanced two solutions: one about windbreaks and the other about irrigations. The advantages of planted agricultural windbreaks, synthesized by Costachescu et al. [100] based on Romanian literature, mentioned, for example, the improvement of microclimatic conditions by modifying the albedo; the decrease by 1–4 °C of the magnitude of daytime air temperature and by 1–2 °C of the annual temperature; reduction of wind speed by 31–55 % within the protected area and by 10–15 % within the exposed part; decrease of unproductive evapotranspiration by up 30 %; increase of air humidity in the proximity of the soil surface by 3–5%; benefits for the neighboring crops in terms of improving growth and development conditions (up to a distance equal to 20–30 times the height of the windbreaks in the sheltered area and 5–12 times the height of the curtain in the exposed part). Therefore, considering the risks of implementing a non-resilient irrigation system, the most viable solution would be windbreaks if there is always a correlation between the ecological requirements of the tree species and the ecological characteristics of the soils [100,103].

### 5.3 The IIAP decision-making process for agricultural land

van Bruggen et al. [37] believe that participatory approaches become “transformative” when they stimulate people to deliver transformative policy processes and intervention plans. Key informants suggested that paving agricultural roads would be extremely useful to ensure that the IIAP decision-making process achieves its “integrative” character. This improvement would make it easier to access the farm land and benefit the entire community. In addition, the reduction and exemption from taxes (agricultural and other taxes) for a certain period could economically strengthen the community that faces material shortages. A consensus was reached on these two measures that could create value for the community in the long term.

The “inclusive” character emphasizes that local councilors represent the community at the town hall level, but there is a gap between central/county authorities and local ones. A key informant mentioned the challenges with bureaucracy and political differences that hinder community development projects. Another participant highlighted disparities in funding based on political affiliations. The central administration is criticized for not listening to local needs, with a plea for better communication.

Concerns were raised about the effectiveness of individual-level campaigns that promote prudent pesticide use since impoverished individuals prioritize food security over potential pesticide risks. Participants suggested incentivizing large agricultural associations to reduce pesticide use. At the individual level, they advocated for tax exemptions to afford healthier products. Delays in receiving aid for crop losses intensified mistrust, prompting small farmers to lease their land to larger associations. Participants suggested creating a dedicated department within city hall to address small farmers’ concerns, highlighting the need for such a department due to the dissolution of the Agricultural Directorate and the limited support provided by the Agency for Payments and Intervention in Agriculture. For the “pluralist character”, social aids help people to cope with economic hardship. One community member noted that, despite the presence of disadvantaged individuals, they do not feel marginalized because the community supports each other by sharing resources and information, such as job opportunities.

The authors synthesized the responses of the key informants into six measures that met the IIAP criteria. These were “Tax exemptions” (for “integrative” and “adaptive” decision-making); “Paved agricultural roads = better access = infrastructure” (for “integrative” character); “Consideration of local needs by the central/county authorities” (“inclusive character)”; “City Hall department for supporting small farmers” and “Incentive to reduces pesticides use for large agricultural associations (for “adaptive” decision-making)”; and “Social aids” (“pluralist” character). These measures are reflected under similar names in other studies related to LD.

For example, on the one hand, considering the economic causes of LD, tax and credit policies are often able to influence the dominance of wealthier farmers and speculative investments in the land [104]. On the other hand, economic incentives for sustainable land use could increase farmers' interest in maintaining and restoring soil quality [105]. The Sustainable Use Directive (Directive 2009/128/EC) asked for compliance with the basic guidelines of integrated pest management (IPM) for all professional users of plant protection products [106]. However, farmers can consider IPM risky due to the novelty of IPM strategies, knowledge, or experience gaps [106]. Therefore, in addition to information campaigns and regulations, incentive-based instruments can influence farmers' decisions about crop protection [106]. Another participatory approach [107] on perceived vulnerability stressors pointed to inadequate road infrastructure that impedes access to agricultural plots during the rainy season, thus weakening the bargaining power of local farmers in Ghana. The lack of road infrastructure has often been considered a source of economic and social vulnerability [20,108]. Kapović Solomun et al. [109], who focused on stakeholders’ perception of the institutional and policy structures involved in LD in Bosnia and Herzegovina, identified the lack of communication and mutual coordination as the main challenges in LD.

*Limitations and future research directions*. Despite the growing literature on case studies that implemented various forms of CBCEA, such as community-based system dynamics, in health, education, and different environmental areas, there is no “uniform”, “evidence-based” “protocol” to implement such an approach for agri-environmental studies. Therefore, there is no “right” way to conduct a participatory process to stimulate transformative change [37], which can determine that certain aspects were omitted. The CBCEA becomes even more difficult due to the impossibility of overlapping experiences and results from other cases due to different societal practices, human behavior, and ecological, economic, and cultural determinants. Next, we designed the workshops to accommodate the key informants’ schedules. At times, exploring certain variables that warranted more detailed discussion was challenging – such as the relationship between “Lies = destructed community”, “Alcohol”, and other elements of the model. Additionally, other causal-effect diagrams could have been identified beyond the existing ones. Due to the multitude of diagrams, attention could not be paid to revealing specific solutions to all causes and effects, but rather to identifying common solutions with the potential for the most significant impact.

Given these limitations, future research should address these challenges and further refine the application of CBCEA in agri-environmental studies. Consequently, they should prioritize exploring the scalability and adaptability of CBCEA in various agri-environmental contexts to determine its effectiveness in various settings. A deeper analysis of the specific variables and their interrelations could provide comprehensive insights into complex systems. Further studies could also investigate tailored solutions to different causes and effects of land degradation, considering unique local conditions and cultural practices. This approach would enhance the ability to design more effective and context-specific interventions to achieve transformative change in sustainable land management.

## 6 Conclusions

The present study is based on a qualitative participatory system dynamic modeling process, which is essential to understanding the role of LD as a source of vulnerability in Romania. A key novelty of this research is the application of CBCEA, which prioritizes the systems thinking potential of key informants. This innovative approach enabled the creation of qualitative causal-effect diagrams, providing an in-depth representation of the system structure related to LD. By adopting a systems thinking approach, the study highlights the complex interaction among social, economic, and environmental factors that influence LD. Furthermore, it offers clear and actionable policy recommendations that can locally and nationally guide sustainable land management strategies. By addressing existing knowledge gaps, the research provides a holistic understanding of the contributing factors of 10.13039/100031136LD, ultimately supporting the development of more effective and sustainable management practices.

The objectives of the study are intricately linked with its results, aimed at comprehensively understanding and addressing the complex issue of land degradation (LD) through a participatory and systems-thinking approach. The objective of identifying direct and indirect causes and effects of LD was achieved through an inclusive and participatory process, where key informants shared valuable insights into the factors that contribute to LD. This process led to the creation of twelve causal-effect diagrams, which illustrate the interconnectedness of socio-economic and environmental elements that influence LD. These diagrams serve as a visual and analytical representation, providing a comprehensive understanding of the structure of the system related to LD and highlighting the complex dynamics at play. Furthermore, the objective of developing qualitative causal-effect diagrams was achieved, effectively mapping out the relationships between various variables. This was an important outcome, as it enabled a detailed examination of how different factors are interlinked. Moreover, the objective of identifying actionable and contextually relevant measures for sustainable land management was fulfilled. The study pinpointed key strategies such as “Windbreaks construction” and “Resilient Irrigation System,” along with specific policy recommendations such as tax exemptions and infrastructure improvements. These findings are designed to promote sustainable land management practices and facilitate transformative change in agricultural land decision-making processes.

In the following, the conclusions of this study encapsulate findings drawn from the comprehensive exploration of the dynamics of LD and offer nuanced policy suggestions with broader national implications.

i) Emphasizing systems thinking: Systems thinking is needed to address complex interconnections between the various components of social and natural systems. Central to our findings is the pivotal role of CBCEA in revealing the intricate socio-economic-ecological dynamics within a particular rural community in Romania. CBCEA facilitated a deeper understanding of local contexts, empowering participants to identify transformative pathways toward effective governance tailored to their needs. This approach underscores the necessity of adopting system thinking in policy-making to address LD comprehensively.
ii) Identifying key interventions: Our analysis revealed twelve causal-effect diagrams, highlighting the interconnectedness of various factors influencing LD in Romania. In particular, “Windbreaks construction” and “(Resilient) Irrigation system” emerged as contextually significant measures with the potential to shape these causal pathways. However, we recommend caution against the uncritical adoption of irrigation systems due to their potential to inadvertently lock systems into an undesirable state if they are not carefully managed and adapted to local ecological conditions.
iii) Local-level policy implications: To catalyze transformative change, straightforward policy suggestions at the local level include “Tax exemptions,” “Social aids,” and establishing a “City Hall department for supporting small farmers.“. These measures are designed to support rural livelihoods and improve ecosystem services, addressing the specific needs identified by community participants. Similarly, initiatives such as “Consideration of local needs by central/county authorities” and “Paved agricultural roads = better access = infrastructure” can promote more inclusive decision-making processes in Romania, ensuring that policies are responsive to local realities.
iv) National-level policy implications: On a larger scale, the implementation of these measures has significant implications for Romania's national-level development strategies. By fostering resilient agricultural practices and improving rural infrastructure within Romania, policymakers can advance broader national goals related to food security, adaptation to climate change, and sustainable development tailored to the local context. Integrating locally tailored policies within the national framework can enhance the resilience and sustainability of agricultural systems throughout Romania, contributing to a more robust and adaptive governance structure.

From a practical perspective, by embracing these insights and recommendations, policymakers in Romania can create a path toward transformative governance that benefits both local communities and the nation as a whole.

## Data availability statement

[Verbatim transcription of workshop, Romanian language], data have been deposited at Zenodo repository (BIOTraCes community): https://zenodo.org/records/11396062, DOI 10.5281/zenodo.11396061.

## Ethics and Consent

The Scientific Council of the Babes-Bolyai University of Cluj-Napoca issued the Research Ethics approval, no 92/January 06, 2023.

## CRediT authorship contribution statement

**Ruxandra Malina Petrescu-Mag:** Writing – review & editing, Writing – original draft, Visualization, Validation, Supervision, Software, Project administration, Methodology, Investigation, Funding acquisition, Formal analysis, Data curation, Conceptualization. **Tibor Hartel:** Writing – review & editing, Writing – original draft, Validation, Methodology, Conceptualization. **Kinga Olga Reti:** Writing – review & editing, Methodology, Conceptualization. **Cornel Mocanu:** Methodology, Investigation, Conceptualization. **Ioan Valentin Petrescu-Mag:** Methodology, Investigation, Conceptualization. **Vlad Macicasan:** Conceptualization. **Dacinia Crina Petrescu:** Writing – review & editing, Writing – original draft, Validation, Methodology, Formal analysis, Conceptualization.

## Declaration of competing interest

The authors declare that they have no known competing financial interests or personal relationships that could have appeared to influence the work reported in this paper.

## Figures and Tables

**Fig. 1A:**
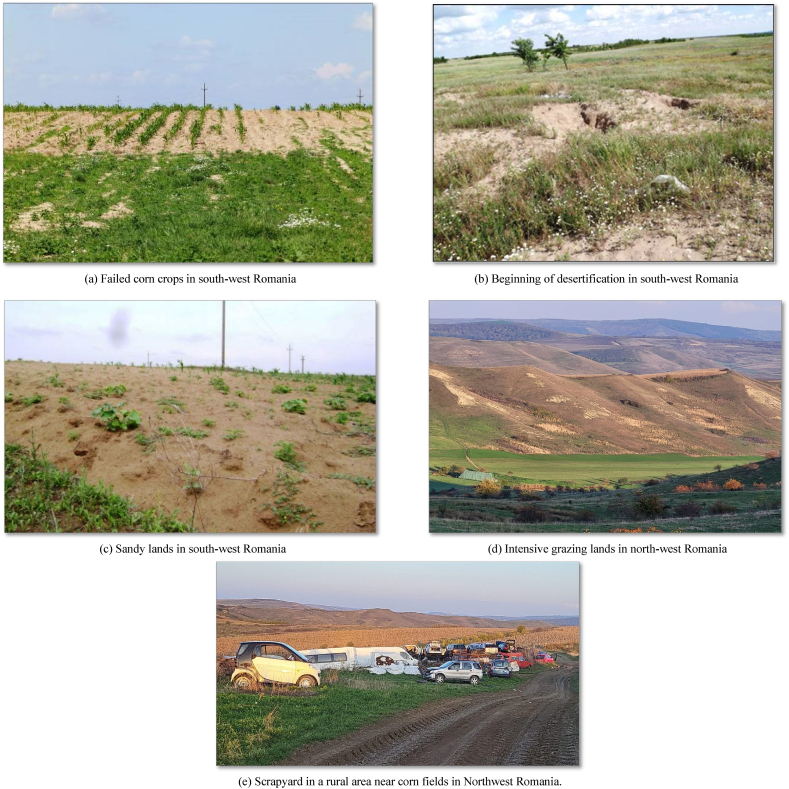
Soils with low resilience in Romania

Source: Ion Patrutoiu personal archive for (a), (b), and (c); authors' personal archive for (d) and (e) (Ruxandra Malina Petrescu-Mag and Dacinia Crina Petrescu)
